# ATon, abundant novel nonautonomous mobile genetic elements in yellow fever mosquito (*Aedes aegypti*)

**DOI:** 10.1186/1471-2164-13-283

**Published:** 2012-06-27

**Authors:** Guojun Yang, Amy Wong, Rebecca Rooke

**Affiliations:** 1 Department of Biology, University of Toronto Mississauga, SB3058, Mississauga, ON, L5L 1 C6, Canada; 2 Cell and System Biology, Toronto, ON, Canada

**Keywords:** Transposable elements, Helitron, Terminal inverted repeats (TIRs), *Aedes aegypti*, Miniature inverted repeat transposable elements (MITEs)

## Abstract

**Background:**

Mosquitoes are important pathogen vectors affecting human and other animals. Studies on genetic control of mosquito mediated disease transmission gained traction recently due to mosquito transgenesis technology. Active transposons are considered valuable tools to propagate pathogen resistance transgenes among mosquitoes, rendering the whole population recalcitrant to diseases. A major hurdle in this approach is the inefficient remobilization activity after the integration of heterologous transposon vectors bearing transgenes into chromosomes. Therefore, endogenous active transposons in mosquito genomes are highly desirable.

**Results:**

Starting with the transposable element database of the yellow fever mosquito *Aedes aegypti* genome, detailed analyses of the members of each TE family were performed to identify sequences with multiple identical copies, an indicator of their latest or current transposition activity. Among a dozen of potentially active TE families, two DNA elements (TF000728 and TF000742 in TEfam) are short and nonautonomous. Close inspection of the elements revealed that these two families were previously mis-categorized and, unlike other known TEs, insert specifically at dinucleotide “AT”. These two families were therefore designated as ATon-I and ATon-II. ATon-I has a total copy number of 294, among which three elements have more than 10 identical copies (146, 61 and 17). ATon-II has a total copy number of 317, among which three elements have more than 10 identical copies (84, 15 and 12). Genome wide searches revealed additional 24 ATon families in *A. aegypti* genome with nearly 6500 copies in total. Transposon display analysis of ATon-1 family using different *A. aegypti* strains suggests that the elements are similarly abundant in the tested mosquito strains.

**Conclusion:**

ATons are novel mobile genetic elements bearing terminal inverted repeats and insert specifically at dinucleotide “AT”. Five ATon families contain elements existing at more than 10 identical copies, suggesting very recent or current transposition activity. A total of 24 new TE families with nearly 6000 copies were identified in this study.

## Background

Transposable elements (TEs) are repetitive sequences found in nearly every eukaryotic species and contribute to a large proportion of the genome in many species. Accurate characterization and categorization of repetitive elements in a genome can be challenging, sometimes significant underestimated in early attempts even in sequenced genomes and previously undiscovered elements continue to emerge. For example, the *Drosophila melanogaster* genome was thought to contain 6-8% TEs, it was soon revised to ~22% [1-3]. Human genome was believed to contain about ~50% repeats, a recent study suggested that they may contribute up to two thirds of the genome [4].

In addition to the fundamental knowledge brought about by research on TEs, the disruptive forces of TEs can be harnessed and utilized to benefit scientific research and technology development. These TE derived tools fall into different application categories. TEs can be used to create stable transgenic organisms that are essential for studies on insertional mutagenesis, enhancer trapping and gene trapping [5]. Due to their disruptive nature, TEs are capable of inserting into multiple sites of a gene, creating a series of TE tagged mutation lines [6]. Different insertional mutant lines may have different levels of severity in terms of phenotypic changes. DNA-type elements are unstable and their excision from an insertional mutant line may restore expression of the disrupted genes, resulting in reversion from mutant phenotypes. These revertant lines provide support for the causative genotype for a phenotype [7]. The target genes in TE tagged lines can be cloned based on the TE tag sequences, a major advantage over the random point mutagenesis approaches (e.g. EMS and UV mediated mutation). Development of this technology for a wide range of hosts requires deep understanding of transposition mechanisms of a variety of elements. The abundance of TEs in most large genomes also make them candidates as genetic markers [8,9]. Gene therapy to treat genetic diseases and cancers using viral vectors encountered significant challenges due to immune response to the viral vectors [10,11], leading researchers to consider DNA transposons for alternative vectors [12]. It has also been demonstrated that PiggyBac transposons can be used to create marker free induced pluripotency stem cells [13]. Genetic control of pest insect population traditionally uses radiation for mass production of sterile male insects. With the insect transformation technology to create transgenic insects bearing genes that can be used for their control, transposons gained much attention to be potential gene drive vectors to spread transgenes among pest populations to achieve genetic control [14].

Yellow fever mosquito, *Aedes aegypti*, is a vector for Yellow Fever, Dengue and Chikungunia virus. Large scale mosquito control has been proven a challenging task. Recently, using biotechnology in mosquito control such as Oxitec has garnered much attention and is currently under pilot test [15]. In this approach, a large number of transgenic male mosquitoes are released to outcompete wild male mosquitoes to mate with female mosquitoes. The transgene causes larva lethality in the next generation. To achieve continuous control effect, a massive number of mosquitoes need to be released on a regular basis. In addition, the ecological consequences, due to the changed mosquito population size and structure, remain a concern. Therefore, cost efficient approaches that introduce minimal alteration of the ecosystem are desirable. One attractive approach is population replacement where a small number of transgenic mosquitoes resistant to pathogens can spread the transgenes to the wild population, pushing the defense line beyond the disease vectors. A critical factor in this approach relies on a gene drive system to spread transgenes. Transposons can increase their frequency in a population, therefore are considered good candidates for a gene drive system [14]. Transgenic mosquitoes resistant to pathogens such as *Dengue* virus and *Plasmodium falciparum* have been established [16,17]. However, the commonly used transposons for insect transgenesis rarely remobilize efficiently once they integrate into the mosquito chromosomes [18-22]. Therefore, endogenous active transposon elements in the mosquito genome may provide a much needed tool.

TEs comprise 47% of the genome of *Aedes aegypti*, a rich source of materials to search for active TEs. TEfam, a database dedicated for vector insects, contains a total of 1089 *A. aetypti* TE families, 826 characterized as retro element families and 247 DNA element families. Many of these families are non-autonomous elements that depend on their autonomous partners to provide transposase for their mobilization. For example there are 143 MITE families that account for about 16% of the total genome sequences [23]. The availability of these TE sequences provides an opportunity to identify candidates for active endogenous TEs in the yellow fever mosquito genome. Previous reverse genetic approaches for active TE discovery suggested that the presence of multiple identical copies of an element is a flashing indicator for current or very recent transposition activity [24,25].

In this study, genome wide analyses were performed to identify elements with multiple identical copies inserted at difference loci. Further analysis of the best candidates for active transposons led to the identification and characterization of 26 families (~6000 copies) of novel transposable elements designated as ATons. These elements are non-autonomous bearing terminal inverted repeats and insert specifically at dinucleotide “AT”, a feature not seen in known TE superfamilies. The autonomous elements for ATons remain mysterious despite exhaustive genome wide database searches.

## Results and discussion

### Identification of ATon-I and ATon-II families in A. Aegypti

**Figure 1:**
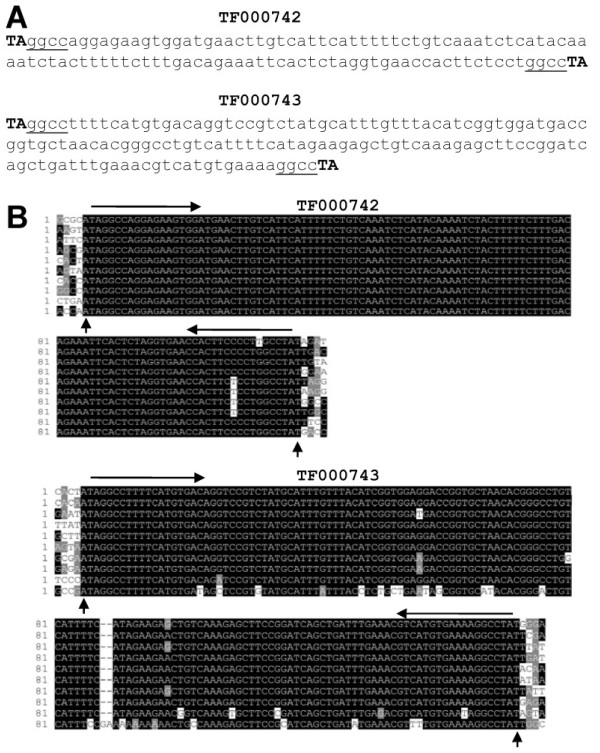
** Repeat families TF000742 and TF000743 in the*A. aegypti*genome.** (**A**) Representative sequences of TF000742 and TF000743 in TEfam. The dinucleotides “**TA**” in bold were annotated as TSD sequences. Underlined nucleotides, shared terminal sequences between TF000742 and TF000743. (**B**) Alignments of 10 randomly picked members of each repeat family. Five nucleotides beyond the dinucleotides “TA” are shown. Horizontal arrowheads, TIRs; upward arrowheads, conserved “A” and “T” beyond the dinucleotides “TA”.

The genome of *A. aegypti* has a high TE content particularly rich in MITEs [26]. In an effort to understand the evolution and activity of TEs in this genome, searches for elements that have multiple identical copies located in different flanking sequences were carried out. All elements that bear complete ends, referred as complete elements, from the 1089 TE families in TEfam were retrieved using MAK [27,28]. The sequences in each family were used to identify identical copies. Out of the 1089 TE families, 73 sequences from 29 families have at least five identical copies (data not shown). A total of 24 sequences from 12 families have at least 10 identical copies in the genome (Table 1). Eight of the 24 sequences are retro elements from three families. Among these, the tSINE family TF000574 is particularly rich in identical sequences with four having more than 10 identical copies: 100, 64, 39 and 10, out of the total copy number of 279. Sixteen of the 24 sequences are DNA elements from nine families. Three DNA TE families, TF000728, TF000742 and TF000743, are particularly rich in identical sequences. TF000728 has one sequence with 116 identical copies out of 257. TF000742 has three sequences with identical copies of 146, 61 and 17. TF000743 has three sequences with identical copies of 84, 15 and 12 (Table 1). All of these three families were categorized in TEfam as MITEs with a target site duplication sequence of “TA” (Figure 1A).

**Table 1:** TE families containing elements existing with more than 10 identical copies

**TEfam ID**	**Family Copy Number**	**Group**	**Number of Identical Copies**	**TE Type**
TF000572	610	a	11	tSINE
		b	18	
		c	11	
TF000573	507	a	13	tSINE
TF000574	279	a	100	tSINE
		b	39	
		c	64	
		d	10	
TF000580	289	a	25	mTA MITE
		b	22	
TF000672	112	a	11	m3bp MITE
		b	14	
TF000708	270	a	25	m8bp MITE
TF000726	237	a	14	m9bp MITE
TF000728	257	a	116	mTA MITE
TF000730	258	a	10	mTA MITE
TF000738	318	a	18	mTA MITE
		b	14	
TF000742	294	a	146	ATon-I
		b	61	
		c	17	
TF000743	317	a	84	ATon-II
		b	15	
		c	12	

* The sequences for these entries can be found in the Additional file 4: sequence file.

For further analyses, the complete copies for each of the three MITE families were retrieved with their flanking sequences. When the sequences from each family were aligned, the target site duplication of dinucleotide “TA” for TF000728 was confirmed, but not for TF000742 and TF000743. In these two families, the sequence conservation extended to an additional nucleotide on each end: the 5’ an “A” and the 3’ end a “T” (outside of the dinucleotide “TA”) (Figure 1B).

**Figure 2:**
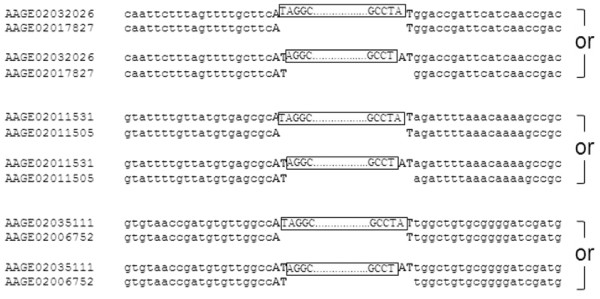
** Representative related empty sites (RES) of ATon-I members.** The top of each pair of sequences contains an ATon-I member and the bottom sequence is a corresponding RES sequence that does not contain the ATon. The target nucleotides “A” and “T” are shown in bold. The accession number for the contig bearing each displayed sequence is shown on the left. Two scenarios are shown: one with the insertion of an element between “A” and “T” (top) and the other with the duplication of “AT” upon insertion of an element (bottom).

TEs can occasionally insert into other repetitive sequences. To identify the junction between the elements in TF000742 and TF000743 families and their flanking sequences, if present, the related empty sequences (RESs) of the flanking sequences were retrieved and aligned. It was found that a dinucleotide “AT” was present in the TE insertion site on the RESs (Figure 2). However, whether the dinucleotide “AT” is duplicated upon insertion or an element inserts between “A” and “T” is not clear. The dinucleotide “TA”, which is observed in the RESs for *Stowaway*-like MITEs, was not observed in the RESs. The specific integration of the TEs bearing terminal inverted repeats at dinucleotide “AT” is a novel feature, therefore they were designated as ATon for “AT” specific insertion transposons. The repeat families represented by TF000742 and TF000743 were subsequently termed ATon-I and ATon-II.

### Features of ATon-I and ATon-II

ATon-I and II elements are 112 and 144 bp respectively. Conceptual folding of these ATon elements revealed that they bear TIRs of 15 and 17 bp (Additional file 1: Figure S1). While the terminal sequence of “ATAGGCC” is conserved between ATon-I and II, the internal sequences of these two families do not share significant similarity. Although a putative stem-loop structure is present in the subterminal regions on both 5’ and 3’ ends of ATon-I, no such structure is apparent in the subterminal regions of ATon-II. Within the sequenced *A. aegypti* genome, there are 294 and 317 complete copies of ATon-I and II respectively. RESs can be found for approximately half (133 for I and 84 for II) of these elements.

Despite the observation that the vast majority of “AT” insertion target sites in the flanking sequences of ATon elements were intact, aberrant empty sites that do not contain intact target sites were found from analyses of all the RES sequences (Additional file 2: Figure S2). They can be grouped into the following four categories: [1] completely missing the dinucleotide “AT”; [2] partially missing the dinucleotide “AT”; [3] deletion beyond the dinucleotide; [4] presence of additional nucleotides that are not present on the flanking sequences of ATon. These imperfect RES sequences are rare (<1%) among the total number of RES sequences for the ATons. These aberrant empty sites are often the only mutations over a long stretch of flanking sequences (up to 1 kb), suggesting that they may have resulted from the transposition activity of ATons.

### Recent expansion of ATon-I and ATon-II families

**Figure 3:**
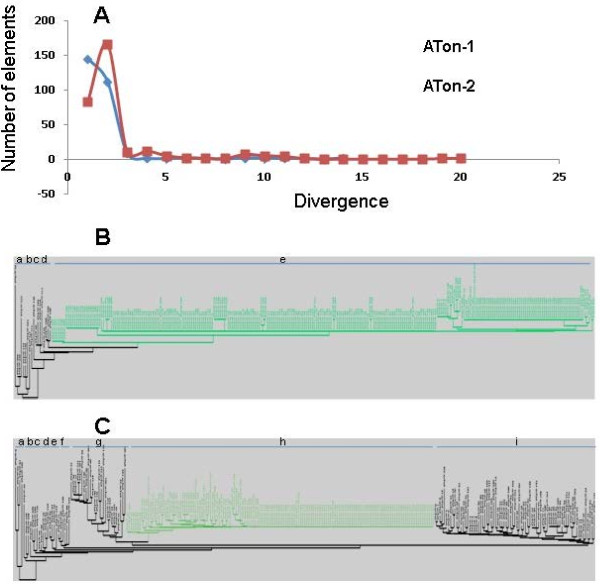
** Amplification of ATon-I and ATon-II families.** (**A**) Divergence distribution of ATons. x-axis, divergence of an ATon member from its consensus sequence; y-axis, number of ATon elements. (**B**) Phylogenetic tree of ATon-I family. Letters “a” to “e”, five subclades. (**C**) Phylogenetic tree of ATon-2 family. Letters “a” to “i”, nine subclades. Green highlights, subclades with large numbers of identical elements.

The large copy number of an ATon family allows the estimation of its proliferation history. When a TE family is amplified from a few original copies, the relative age of each element can be measured by the level of sequence divergence from the consensus sequence. The sequence divergence for each ATon element from its corresponding family consensus sequences was calculated. The number of elements of a defined sequence divergence value was plotted against sequence divergence values to obtain relative proliferation time for each ATon family (Figure 3A). For ATon-I, 146 elements have identical sequences (0% divergence) compared to the consensus sequence of the family and 112 copies have divergence values between 0-1%. Only 20 copies exist at divergence values ranging from 2% up to 13%. In ATon-II, 83 elements have sequences identical to the consensus sequence and 166 copies have a divergence value ≤ 1%. Forty seven copies exist at divergence values between 1% and 10%. Three copies exist at divergence values from 10% to 20%.

To understand the evolution of the ATon families, phylogenetic analyses for the two ATon families were performed. The elements in ATon-I family can be grouped into five subclades (Figure 3B). Subclades “a” to “d” each contain a few elements. In contrast, subclade “e” is large and contains many identical element copies. Similarly, the elements in ATon-II can be grouped into seven subclades (Figure 3C). Subclades “a” to “g” each contain a few elements that show minimal sequence similarities. Subclade “i” contains 75 copies that demonstrated some relatedness to other members in the clade whereas subclade “h" contained the largest number of elements with several identical copies (indicated by no phylogenetic divergence). The phylogenetic analyses suggested that the high copy numbers for these elements are due to recent amplification of these elements a few ancient copies.

### Distribution of ATon-I in different strains of A. Aegypti

**Figure 4:**
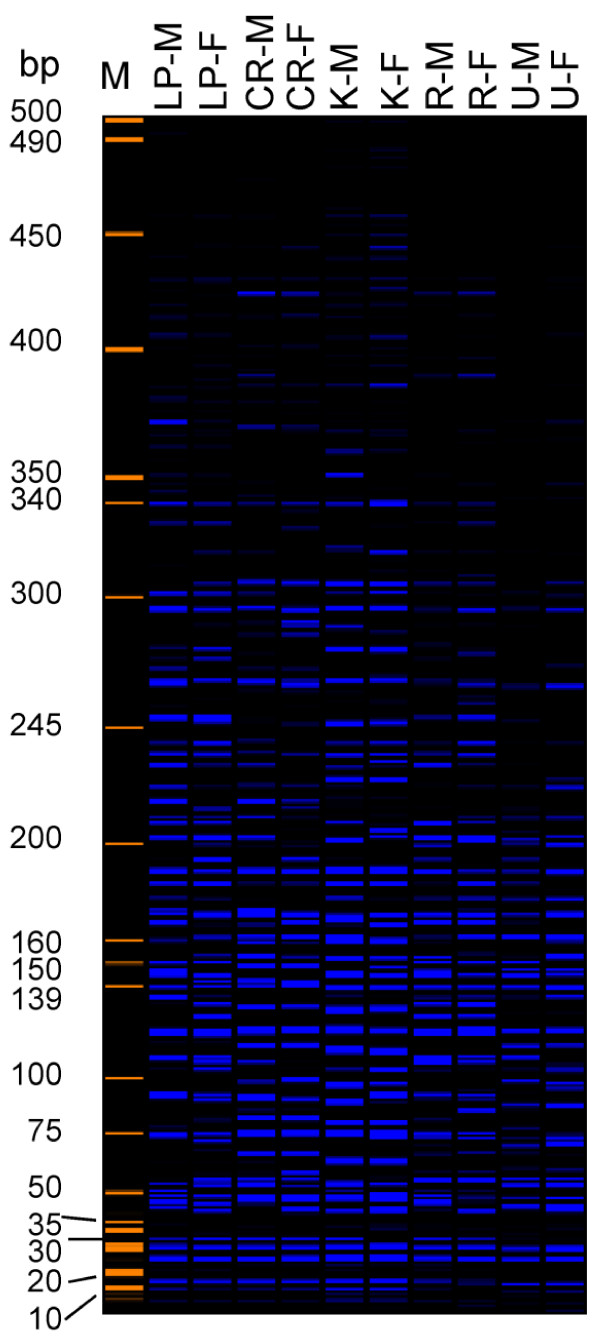
** Transposon display of ATon-I in different strains of*A. aegypti.*** M, size marker; A female (F) and male (M) individual are shown for each strain. LP, Liverpool; CR, Costa Rica; R, Rockefeller; K, KHW; U, UGAL. The selective base “C” was used for selective amplification.

There can be differential accumulation of various TE families in different strains or ecotypes of a species. For example, different lines of the same rice cultivar contain dramatically different copy numbers, from 50 to ~1000, of the active MITE family *mPing*[29]. Since ATon-I family appears to have amplified very recently in the sequenced Liverpool strain of *A. aegypti* which was collected in 1936 from West Africa, it is interesting to know whether this ATon family is similarly distributed in *A. aegypti* mosquito strains collected from other locations. In the Americas *A. aegypti* was first introduced from Africa during European exploration and colonization in the late 1500 s thus providing at least four centuries of separation. The distribution of ATon-I family in mosquito strains collected from different locations in the Americas was surveyed (Figure 4). These strains included Costa Rica (collected in 1998 from Costa Rica), UGAL (collected in 1990s from Georgia USA) and Rockefeller (collected in ~1950 from Antilles). KHW is a white-eyed mutant line of Rockefeller. The copy numbers of ATon-I in these strains of *A. aegypti* were estimated using transposon display, a modified version of amplified fragment length polymorphism (AFLP) (Vos et al. 1995). When one selective base (“C”) was used to reduce the total number of bands in each lane, approximately 70 bands were visible on each lane of the Liverpool strain, consistent with the expected results (approximately one quarter of the total genomic copy number of 294 in the sequenced inbreed Liverpool strain LVP^1b12). Despite the fact that the Liverpool mosquitoes used in this study underwent at least 13 consecutive generations of single pair inbreeding, there are still about 25 (~18%) bands have different sizes between the two individuals. *A. aegypti* has homomorphic sex chromosomes with a small sex-determining region on chromosome 1, therefore the different sized bands are probably not caused solely by the different sex of the two individuals. The other strains showed a similar number of bands to that of the Liverpool strain and a similar level of polymorphism between two individuals. These results suggest that ATon-I has amplified to a similar copy number in all of the tested strains of *A. aegypti*.

### Abundant new ATon families in A. Aegypti genome

**Figure 5:**
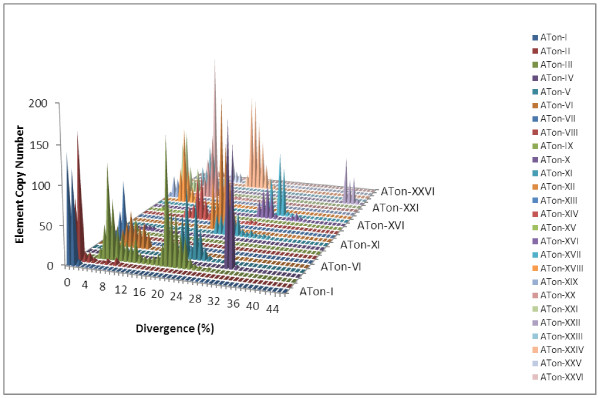
** Amplification of the 26 ATon elements in*A.aegypti.*** The divergence of each copy form the consensus sequence is indicated on the x-axis while the copy number is indicated on the y-axis.

To find out how abundant ATons are in the *A. aegypti* genome, exhaustive genome wide searches were performed to identify repetitive elements that contain terminal inverted repeats flanked by nucleotides “A” and “T”. The RESs, when exist, were retrieved to confirm the target site dinucleotide “AT”. A total of 26 ATon-like families were identified, including the two families described above. Interestingly, all of these elements have the nucleotide “T” internal to the “A” on the 5’ end and an internal “A” to the “T” on the 3’ end (Additional file 3: Table S1). The additional 24 ATon families contributed to a total 5867 copies. Sequences in each of the 24 families are more diverged than those in ATon-I and ATon-II (Figure 5). Nevertheless, three families have an element with more than 10 identical copies: ATon-XII with 17 out of 270, ATon-XXII with 15 out of 248 and XXIV with 49 out of 346, suggesting that these elements were recently or may be currently active (Additional file 4: Sequence File).

**Figure 6:**
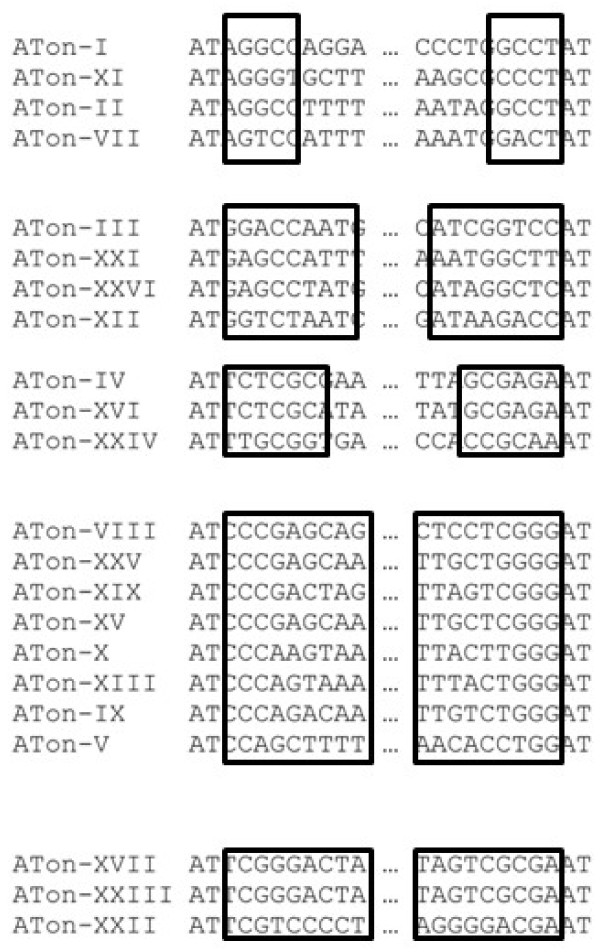
** Grouping of ATon families in*A.aegypti.*** Boxed regions, similar terminal sequences; Dots, omitted internal sequences.

While each ATon family has a unique internal sequence and TIR sequence, weak sequence similarity exists at the extreme ends of TIRs among some families (Figure 6). Based on this sequence similarity, five groups that account for 22 ATons families can be identified. The length of the similar regions ranges from 5 to 9 nucleotides. To investigate whether ATons are transcribed, ESTs containing ATon sequences were retrieved from databases. Of the 26 families, 23 resulted in EST hits. The number of hit sequences varied from 1 to 40 among the families (Additional file 3: Table S1). Since ATons do not encode proteins, their presence on ESTs in the untranslated regions resulted from the cotranscription with the genes (not shown) they are located in.

### Comparison of ATons with helitrons and MITEs

ATons lack protein coding capacity and their transposition is dependent on transposases produced by autonomous elements. Identification of putative transposase sources for ATons will shed light on their transposition mechanisms and facilitate their classification. The autonomous elements of DNA TEs are typically expected to bear similar TIRs, to the non-autonomous elements they mobilize. Occasionally, these similarities can be very limited; consequently, identifying the autonomous partner is more challenging. Extensive searches for large DNA fragments flanked by similar terminal sequences to those of ATons were performed. All of the genomic DNA fragments from 500 bp to 200 kb flanked by the termini (>10 bp) of ATons were retrieved and analyzed. Close inspection of these large fragments did not yield conclusive clues to the putative transposase for ATons.

Since ATon elements, like *Helitrons*, are always flanked by an “A” on the 5’ end and a “T” on the 3’ end, it is possible that ATons are related to *Helitron* elements that insert between “A” and “T”. However, there are marked difference between ATons and reported *Helitron*s. The latter have conserved terminal motifs of 5’ “TC” and 3’ “CTRR”, although ATons have a “T” internal to the 5’ “A”, only 8 families have a “C” after it [30]. *Helitron*s were originally thought to bear subterminal palindromic sequences, a number of elements found in fungi, sea urchin, sea anemone and *Drosophila* are often palindrome free [31-33]. The presence of subterminal palindromic motifs on some ATons resembles that on some *Helitron*s. *Helitron*s typically do not bear TIRs. However, it has been reported that the maize genome contain Heltir sequences that have terminal inverted repeats resembling *Helitron* 3' termini [34]. To see whether there are autonomous *Helitron*s in *A. aegyypti* genome that bear the TIR sequences of ATons as a terminus, genomic sequences of 20 kb near ATon TIR sequences were analyzed, nevertheless no *Helitron* protein domains (Rep, Hel, EN or OUT) were found in these sequences.

On the other hand, ATons share some similarity in features to MITEs, they are quite small (<500) and have TIRs. As described in Results, the dinucleotide “AT” may also be considered as a specific TSD sequence. If ATons are MITEs, it is possible that transposase coding autonomous elements bearing the TIR sequences of ATons may exist in the genome. In an effort to find such elements in the whole genome, genomic sequences (up to 20 kb) flanked by ATon TIRs were analyzed. However, no conclusive evidence was found for the presence of such transposase coding elements. A similar scenario occurred for the identity of a recently discovered Drosophila element named DINE-I [35]. However, DINE-I elements do not bear TIRs that are the critical features of MITEs. In addition, DINE-I has a preferred insertion site of “TT” and partial RepHel coding sequences were found to be associated with some elements. These feature led to the proposal that DINE-I elements are *Helitron*s. In contrast, ATons bear TIRs like MITEs and insert at “AT” like typical *Helitron*s. Therefore, the classification of ATons and components of their transposition machineries remain to be determined.

## Conclusion

Genome wide analyses of TEs in *A. aegypti* identified 24 candidate elements from 12 familes for very recent or current transposition activity. Among the best candidate TEs are five ATon families, novel elements bear terminal inverted repeats and insert specifically at dinucleotide “AT”. In this study, 24 previously unidentified TE families with nearly 6000 copies were characterized. However, despite exhaustive search efforts in the genome sequences, the autonomous elements and the classification of ATons remain mysterious.

## Methods

### Database analysis and sequence retrieval

The assembled genomic sequences (version AaegL1) of *Aedes aegypti* were downloaded from Vectorbase [23]. The sequences of the annotated TEs of *A. aegypti* were obtained from TEfam (http://tefam.biochem.vt.edu/tefam/). Sequences of the members of a TE family were extracted from the *A. aegypti* genome database using the MITE Analysis Kit (MAK) [27,28]. Theoretical folding of ATon sequences was performed with Mfold (http://mfold.bioinfo.rpi.edu/). A Perl script named “Identical_Element_Retriever” was used to identify elements with identical copies in the genome (available upon request).

### Phylogenetic and sequence divergence analysis

All retrieved copies of an ATon family were aligned with ClustalX (2.0.12). Phylogenies were constructed with PHYLIP DNAPARS web server at (http://bioweb.pasteur.fr/phylogeny/intro-en.html) with 1000 replicates. The phylogenetic trees were visualized on the TreeDyn 198 web server at (http://www.phylogeny.fr/) [36]. The consensus sequence, based on the alignment described above, was constructed for each ATon family. The divergence value was calculated using the MAK program “Divergence” function. The sequence similarity between an element and the consensus sequence was calculated using BLASTN with manual inspection. Divergence is designated as the complement of similarity. The number of elements with divergence in a certain range was counted and plotted against the divergence values. MAK was also used to obtain the related empty sites (RES) for each ATon family with 50 bp flanking sequences on both ends. RES sites represent sites with sequences similar to the flanking sequences of an ATon but do not bear the element. To understand whether ATons are transcribed in the *A. aegypti* genome, the consensus sequences were used in a standalone BLASTN search against the *A. aegypti* EST database (Vectorebase). Sequences that were longer than 60 bp, had an E-value equal to or less than 10^-6^, and had a similar identity greater than 70% were considered to be significant. Genome wide searches for additional ATon families in *A. aegypti* was automated with a Perl script called ATon_retriever (available upon request) with subsequent manual inspection. Elements with at least 10 full-length copies in the genome were considered to be candidate ATon families for further characterization.

### Transposon display

Transposon display was performed as previously described [37]. The following parameters were used: restriction endonuclease *Bfa*I (NEB), TE preamplification primer: 5’ cagaaaaatgaatgacaagttcatccacttctcctg 3’, and TE selective primers: 5’ caagttcatccacttctcctggcct 3’. Adaptor primer is: GACGATGAGTCCTGAGTAG + selective base(s). The selective primer was labeled with 6’-FAM fluorescent dye (Applied Biosystems) on the 5’ end and PCR products from selective amplification were analyzed on the ABI 3700 genetic analyzer (TCAG, The Hospital for Sick Children, Toronto). The output was analyzed and visualized with Genographer 2.1.4, an upgraded version by Travis Banks from the original program [38].

## Abbreviations

TE, Transposable element; RES, Related empty site; TIR, Terminal inverted repeat; ITR, Inverted terminal repeat; TSD, Target site duplication; LTR, Long terminal repeat; SINE, Short interspersed transposable element; LINE, Long interspersed transposable element; MITE, Miniature inverted repeat transposable element; AFLP, Amplified fragment length polymorphism.

## Competing interests

The authors declare that they have no competing interests.

## Authors’ contributions

GY designed the studies, developed Perl codes, performed analyses and wrote the manuscript. AW performed TE sequence analyses and helped in manuscript draft. RR performed sequence analyses to obtain families containing identical elements and helped in manuscript draft. All authors read and approved the final manuscript.

## Funding

Supported by the National Sciences and Engineering Research Council (NSERC) Discovery Grant (Canada) (RGPIN 371565 to GY), Canadian Foundation for Innovation (CFI24456 and IOF-12 to GY) and Ontario Research Foundation (ORF24456 to GY) and the University of Toronto.

## Supplementary Material

Additional file 1 **Figure S1. **Conceptual folding of TF000742 (A) and TF000743 (B). Stem regions, regions with inverted repeat configuration. A putative stem loop structure is present in each subterminal region of TF000742 on both 5’ and 3’ ends. Click here for file

Additional file 2 **Figure S2.** Four types (I-IV) of aberrant RESs of ATon flanking sequences. Representative RESs are shown for each type. In each set of three sequences, the top sequence contains intact target site nucleotides “A” and “T” at RES, the middle sequence bears an ATon arbitrarily placed between the two nucleotides, and the bottom sequence contains an aberrant targets site. Type I, missing both “A” and “T” of the target site; Type II, missing at least one nucleotide of the target site “AT” and deletions beyond “AT”; Type III, missing both nucleotides of the target site “AT” and deletions beyond the target site; Type IV, presence of additional nucleotides between “A” and “T”. The copy number of each sequence in the genome is shown on the right. Click here for file

Additional file 3 **Table S1.** Summary data for the 26 ATon families in ***A.aegypti*.** Each family is represented by its accession, size, copy number, RESs, and EST hits. Ten terminal nucleotides on both 5’ and 3’ ends are shown for each family. Click here for file

Additional file 4 **Sequence File.** Sequences for each TE element that exists at more than 10 identical copies in the *A. aegypti* genome sequences. Click here for file
