# Supplementary figures and images for: ATon, abundant novel nonautonomous mobile genetic elements in yellow fever mosquito (Aedes aegypti)
Source: BMC Genomics. 2012 Jun 27;13:283. doi: 10.1186/1471-2164-13-283 (PMC3422177; doi:10.1186/1471-2164-13-283)

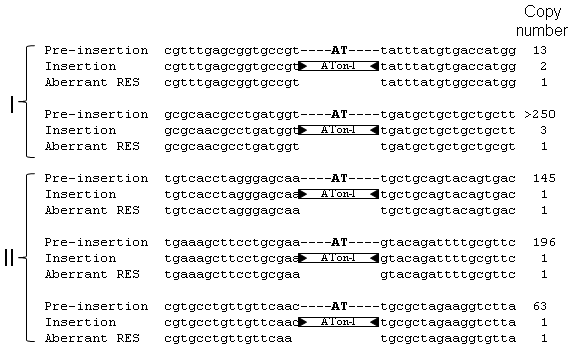
Supplementary Fig. 2

Supplement: Additional file 2 — Figure S2. Four types (I-IV) of aberrant RESs of ATon flanking sequences. Representative RESs are shown for each type. In each set of three sequences, the top sequence contains intact target site nucleotides “A” and “T” at RES, the middle sequence bears an ATon arbitrarily placed between the two nucleotides, and the bottom sequence contains an aberrant targets site. Type I, missing both “A” and “T” of the target site; Type II, missing at least one nucleotide of the target site “AT” and deletions beyond “AT”; Type III, missing both nucleotides of the target site “AT” and deletions beyond the target site; Type IV, presence of additional nucleotides between “A” and “T”. The copy number of each sequence in the genome is shown on the right. [file 1471-2164-13-283-S2.docx]
