# Supplementary material for: ATon, abundant novel nonautonomous mobile genetic elements in yellow fever mosquito (Aedes aegypti)
Source: BMC Genomics. 2012 Jun 27;13:283. doi: 10.1186/1471-2164-13-283 (PMC3422177; doi:10.1186/1471-2164-13-283)
Supplement: Additional file 3 — Table S1. Summary data for the 26 ATon families in A.aegypti. Each family is represented by its accession, size, copy number, RESs, and EST hits. Ten terminal nucleotides on both 5’ and 3’ ends are shown for each family. [file 1471-2164-13-283-S3.docx]

Supplementary Table
