# Supplementary material for: ATon, abundant novel nonautonomous mobile genetic elements in yellow fever mosquito (Aedes aegypti)
Source: BMC Genomics. 2012 Jun 27;13:283. doi: 10.1186/1471-2164-13-283 (PMC3422177; doi:10.1186/1471-2164-13-283)
Supplement: Additional file 4 — Sequence File. Sequences for each TE element that exists at more than 10 identical copies in the A. aegypti genome sequences. [file 1471-2164-13-283-S4.docx]

**Supplementary Sequence File**

***A. aegypti* TE families containing elements existing at more than 10 identical copies**

> TF000572, Group a, 11 identical copies ggggcccagatagccgtagcggtaaacgcgcagctattcagcaagaccaagctgagggtcgtgggttcgaatcccaccggtcgaggatcttttcgggttggaaattttctcgacttcccagggcatagagtatcttcgtacctgccacacgatatacgcatgcaaaaatggtcattggcatagtaagctctcagttaataactgtggaagtgctcataagaacactaagctgagaagcaggctctgtcccagtggggacgtaacgccagaaagaagaagaagaa

> TF000572, Group b, 18 identical copies ggggcccagatagccgtagcggtaaacgcgcagctattcagcaagaccaagctgagggtcgtgggttcgaatcccaccggtcgaggatcttttcgggttggaaattttctcgacttcccagggcatagagtatcttcgtacctgccacacgatatacacatgcaaaaatggtcattggcatagtaagctctcagttaataactgtggaagtgctcataagaacactaagctgagaagcaggctctgtcccagtagggacgtaacgccagaaagaagaagaagaa

> TF000572, Group c, 11 identical copies ggggcccagatagccgtagcggtaaacgcgcagctattcagcaagaccaagctgagggtcgtgggttcgaatcccaccggtcgaggatcttttcgggttggaaattttctcgacttcccagggcatagagtatcttcgtacctgccacacgatatacacatgcaaaaatggtcattggcatagtaagctctcagttaataactgtggaagtgctcataagaacactaagctgagaagcaggctctgtcccagtggggacgtaacgccagaaagaagaagaagaa

> TF000573, Group a, 13 identical copies ggggccttccttagccgagtggttagagtccgcggctacaaagcaaagccatgctgaaggtgtctgggttcgattcccggtcggtccaggatcttttcgggttggaaattttctcgacttccctgggcatagagtatcatcgtacctgccacacgatatacgaatgcgaaaatggcaactttggcatagaaagctctcagttaataactgtggaagtgctcataagaacactaagctgagaagcaggctctgtcccagtgaggacgtcaatgccaagaagaagaa

> TF000574, Group a, 100 identical copies ggggacggacctggtgtagtggttagaacactcgcctctcacgccgaggacctgggatcgaatcccatccccgacatagtcacttatgacgtaaaaagttatagtgacgacttccttcggaagggaagtaaagccgttggtcccgagatgaactagcccagggctaaaaatctcgttaataaagtcaaaccaaccaaccaa

> TF000574, Group b, 39 identical copies ggggacggacctggtgtagtggttagaacactcgcctctcacgccgaggacctgggatcgaatcccatccccgacatagtcacttatgacgtaaaaagttatagtgacgacttccttcggaagggaagtaaagccgttggtcccgagatgaactagcccagggctaaaaatctcgttaataaagtcaaaccaaccaacca

> TF000574, Group c, 64 identical copies ggggacggacctggtgtagtggttagaacactcgcctctcacgccgaggacctgggatcgaatcccatccccgacatagtcacttatgacgtaaaaagttatagtgacgacttccttcggaagggaagtaaagccgttggtcccgagatgaactagcccagggctaaaaatctcgttaataaagtcaaaccaaccaac

> TF000574, Group d, 10 identical copies gggacggacctggtgtagtggttagaacactcgcctctcacgccgaggacctgggatcgaatcccatccccgacatagtcacttatgacgtaaaaagttatagtgacgacttccttcggaagggaagtaaagccgttggtcccgagatgaactagcccagggctaaaaatctcgttaataaagtcaaaccaaccaacc

> TF000580, Group a, 25 identical copies cagtgaaacctccatgagtcgatattgaagggaccatcgactcatggaaatatcgagtcatggaacagcaatcctttggaaagctgtttgaagggaccatcatagtaaccatgaaattttgtttccagtatggttccatgagtcgatatcgagtcatggaacatcgactcatggagggatcactg

> TF000580, Group b, 22 identical copies cagtgaaacctccatgagtcgatattgaagggaccatcgactcatggaaatatcgagtcatggaacagcaatcctttggaaagctgcttctagggaccatcatagtaaccatgaaattttgtttttagtatggttccatgagtcgatatcgagtcatggaacatcgactcatggaggtatcactg

> TF000672, Group a, 11 identical copies cgtgcttattctgcgcggcgtgtgagtcgagacgactcgctctcaccgcgagtgacgtgagacgactaatgttaatcaaatgggagcgagtcgacaattgtcacctcattcgactcgtgtcacctcacacgccgcgcagaataggcacg

> TF000672, Group b, 14 identical copies cgtgcttattctgcgcggcgtgtgagtagagacgagtcgctctcaccgcgagtgacgtgagacgactaatgttaatcaaatgggagcgagtcgacaattgtcacctcattcgactcgtgtcacctcacacgccgcgcagaataggcacg

> TF000708, Group a, 25 identical copies cagggattgaatcctgagaaaattgagagaatctctcacgtatcgctctctgtaactatcataacatgctgcacattatgagagactgtttatcgtatactgctacaagtttgatcagattggggggatagtagtgcatgataaaacccctctaaacatgctccaagcctgggggacactgttgttattggaagttcgtggattgtttatcgtgccagtaaagaaaaacacctatccccaacggtaaacaagcgagaaaaatggattttatcatcgctctctcgttggggctctcgctcgctgcttccatttatcagacttgttacaccttgcgatacaatgacgatcgtggaccgcaacagatgggatgtttttctttgcttgctttgatcgtgcttcattctcaaatgagagcgaattctgcaacgcctg

> TF000726, Group a, 14 identical copies caatgagaatatataccgaggtgaggaagacgacatttagtgtgcgtgacatccgtgtacggtgcaaaatgtttcacaactacaagcgggcgagctcccataagaagccgtgtaaattagtgacgtagttatttttgtttacgttttttctctttactaatacatagccattgcttcttcttccacaccttagtatatattctcattg

> TF000728, Group a, 116 identical copies aagcctgtccacgttaaatttcggacacccaaataatggcagcaaaaaaaagttactcataattcaacaaaaacaattgtttatttcagaataaaagagttatatctacaaaataaacagttgacaaggatgttaatccttctttctgtaaaattctcgaactttctgtggtacacccgccatccgtgtccgaaatttatcgtggacaggctt

> TF000730, Group a, 10 identical copies cacccgattctgtttttgcacgggggatgcgtaccgtgcaaaaaaagttttcagttcaaaatttcaaaaaccgtgcaaaaaaagtaacaccatttctcgacgtttcatgcaaaaataaggttttggcggaaaaaaatgtatggaaactttttttgcacggccgtgtaaaaaaaatccgtgcaaaaacagaatcgggtg

> TF000738, Group a, 18 identical copies cagtggatccacaattaagaatcgtccacaatttatgaatcagctgactttaaatgacaaaataacaacaaaaatcaacacaaaacatcacgtaaacaattttactcaaaataaattataagcgaaaatgtttccgtgatgttttgtgctattttttactgttattttgtcctttaaaggcagctgattcataaattgtgggtgattcttaattgtggattcactg

> TF000738, Group b, 14 identical copies cagtggatccacaattaagaatcgcccacaatttatgaatcagctgactttaaatgacaaaataacaacaaaaatcaacacaaaacatcacgtaaacaattttactcaaaataaattataagtgaaaatgtttccgtgatgttttgtgctattttttactgttattttgtcctttaaaggcagctgattcataaattgtgggtgattcttaattgtggattcactg

> TF000742, Group a, 146 identical copies ggccaggagaagtggatgaacttgtcattcatttttctgtcaaatctcatacaaaatctactttttctttgacagaaattcactctaggtgaaccacttcccctggcc

> TF000742, Group b, 61 identical copies ggccaggagaagtggatgaacttgtcattcatttttctgtcaaatctcatacaaaatctactttttctctgacagaaattcactctaggtgaaccacttcccctggcc

> TF000742, Group c, 17 identical copies ggccaggagaagtggatgaacttgtcattcatttttctgtcaaatctcatacaaaatctactttttctttgacataaattcactctaggtgaaccacttcccctggcc

> TF000743, Group a, 84 identical copies ggccttttcatgtgacagatccgtctatgcatttgtttacatcggtggaggaccggtgctaacacgggcctgtcattttcatagaagaactgtcaaagtgcttcccgatcagctgatttgagacgtcatgtgaataggcc

> TF000743, Group b, 15 identical copies ggccttttcatgtgacagatccgtctatgcatttgtttacatcggtggaggaccggtgctaacacgggcctgtcattttcatagaagaaatgtcaaagtgcttcccgatcagctgatttgagacgtcatgtgaataggcc

> TF000743, Group c, 12 identical copies ggccttttcatgtgacagatccgtctatgcatttgtttacatcggtggaggaccggtgctaacacgagcctgtcattttcatagaagaactgtcaaagtgcttcccgatcagctgatttgagacgtcatgtgaataggcc

>ATon-XII, 17 identical copies

atggtctaatccaccggtgtactaaattcactcggtctgagaattgtgacacttgagcggggcacgctcccgtatgatccccacgtgatctggacccgctctagtgtcaaaattcttcgaccgagtcagtttagtacaccagtggataagaccat

>ATon-XXII, 15 identical copies

attcgtccccttaatgctacaactagataactcgaaaatcaaaattttgagatgtgcaactttgaaagtatgaccgtttaacaaggtgatggttaatcgcagagattatgattgaaaaataatctttaacttgtgtattttgaatcacacgcttaagacctgtggatattttgcagatctaattaagaaaaatgttttgacatattgaagtcaaaaatttcaaatttcgagttatctagttgtagcatcaaggggacgaat

>ATon-XXIV, 49 identical copies atttgcggcggcgacgacgatggcttggacgcttctccgagcagcagtagtttgccgctcggagaagcgcccaagccatcgtcatcgccgccgcaaat
